# Construction and analysis of a nomogram prediction model for post-infectious bronchiolitis obliterans in children with adenovirus pneumonia after invasive mechanical ventilation

**DOI:** 10.1186/s12887-023-03883-9

**Published:** 2023-02-16

**Authors:** Li Peng, Silan Liu, Tian Xie, Yu Li, Zhuojie Yang, Yongqi Chen, Liangji Deng, Han Huang, Xiaofang Ding, Min Chen, Lin Lin, Sangzi Wei, Lili Zhong

**Affiliations:** Hunan Provincial Key Laboratory of Pediatric Respirology, Pediatric Medical Center, Hunan Provincial People’s Hospital (the First Affiliated Hospital of Hunan Normal University), Fu-Rong District, 61 Jie-Fang West Road, Changsha, 410005 People’s Republic of China

**Keywords:** Adenovirus pneumonia, Children, post-infectious bronchiolitis obliterans, Risk factors, Nomogram prediction model

## Abstract

**Background:**

Post-infectious bronchiolitis obliterans (PIBO) is the most common sequelae in children with adenovirus pneumonia (ADVP). However, there are few studies on the risk factors for PIBO occurrence. This study aims to investigate the risk factors for PIBO in pediatric patients with severe ADVP, especially after invasive mechanical ventilation (IMV), as well as to build a nomogram prediction model.

**Methods:**

The clinical data, laboratory and imaging features, and treatment of 863 children with ADVP under 3 years old who were admitted to our hospital from January to December 2019 were retrospectively analyzed. Among them, 66 children with severe ADVP received IMV treatment. The situation and the influencing factors of PIBO in children with severe ADVP were explored, and a nomogram prediction model was constructed.

**Results:**

Among the 863 cases of ADVP, 46 cases (5.33%) developed PIBO. Duration of fever, IMV, complications, and neutrophil percentage were independent risk factors for PIBO in children with ADVP. Among the 66 patients with ADVP who underwent IMV, 33 patients (50.0%) developed PIBO. Gender, duration of fever, adenovirus (ADV) load, and mixed fungal coinfections were independent risk factors for PIBO. In the nomogram prediction model analysis, the area under the curve (AUC) was 0.857; in addition, Hosmer‒Lemeshow (H–L) detection reflected good alignment (χ2 = 68.75, *P* < 0.01).

**Conclusions:**

A nomogram prediction model, which can be utilized to predict PIBO occurrence in pediatric patients with ADVP after IMV at an early time period, was successfully built.

## Introduction

Children under 5 years with pneumonia exhibit high morbidity and mortality [1]. Adenovirus (ADV), which is a type of pathogen, can cause pneumonia throughout the world and accounts for approximately 4–10% of respiratory tract infections in children [2, 3]. Some severe ADV pneumonia (ADVP) patients may have different degrees of respiratory complications and sequelae, among which post-infectious bronchiolitis obliterans (PIBO) is the most common complication [4, 5]. PIBO is a group of chronic airflow obstruction syndromes with repetitive or persistent dyspnea and airflow obstruction as the main manifestations. PIBO mainly manifests as damage to the small airway epithelium and partial or complete obstruction of bronchioles or alveolar tubules by granulation tissue or fibrotic tissue [6].

A previous retrospective study of 415 children with ADVP with PIBO showed that hospitalization for more than 30 days, multifocal pneumonia, and hypercapnia were independent risk factors for PIBO [7]. A 5-year follow-up study of children with ADVP showed that children who developed PIBO had a significantly longer hospital stay, were more likely to be admitted to the ICU, and received invasive mechanical ventilation (IMV) [8]. Moreover, the use of IMV reflects severe lung disease in children; in addition, IMV inevitably causes mechanical lung injury. IMV is an independent indicator for PIBO after infection in children under 3 years [9]. However, we found that not all children with ADVP who underwent IMV developed PIBO during the clinical follow-up. The objective indicators for developing PIBO in pediatric patients with ADVP after receiving IMV have not been investigated. Therefore, children with ADVP who underwent IMV were selected as the research subjects in this study. Their clinical characteristics were summarized, and the risk factors for PIBO were analyzed. Moreover, the screened independent risk factors were further constructed into a nomogram prediction model to assist in the clinical screening of pediatric patients, to formulate preventive strategies, and to improve the treatment effects.

## Methods

### Patients

From January 2019 to December 2019, 863 children who were hospitalized in our hospital, diagnosed with ADVP, and who underwent IMV were selected as the research subjects. The children were under 3 years of age. According to the 2-year follow-up, demographic and clinical variables were compared between the PIBO and non-PIBO groups. The Declaration of Helsinki was followed throughout the duration of the study. Informed consent was obtained from the subjects and their guardians who understood and participated in these experiments. The study was approved by the Ethics Committee of Hunan Provincial People's Hospital with judgment reference number 2020–07. All of the methods were performed in accordance with the relevant guidelines and regulations. 

The diagnostic criteria for ADVP include acute lower respiratory symptoms, lung infiltration, and ADV infection [10]. The exclusion criteria included patients with chronic lung disease, wheezing or a history of asthma, and incomplete case information, among other criteria. The clinical diagnostic criteria of PIBO are as follows [11]. (i) Persistent wheezing, coughing, shortness of breath, dyspnea, or exercise intolerance. (ii) Extensive wheezing and crackles that can be heard in both lungs for > 6 weeks, as well as the lungs responding poorly to bronchodilators. (iii) High-resolution computed tomography (CT) showing PIBO changes (such as a mosaic sign, bronchiectasis, and bronchial wall thickening). (iv) Lung function showing small airway obstructive or mixed ventilatory dysfunction and bronchodilation tests mostly being negative. (v) Cystic fibrosis, congenital bronchopulmonary dysplasia, and other diseases caused by cough and asthma being excluded. 

### Data collection

Clinical information (gender, age, significant comorbidities, signs and symptoms, laboratory characteristics, associated multiorgan dysfunction, and treatment) was collected. The experimental data mainly included routine blood tests, mixed infections, and T-cell differentiation, among other data.

### Real-time PCR (RT‒PCR) detection of ADV in nasopharyngeal swabs

Specialist nurses collected the specimens in our hospital. The length of the throat swab was extended from the earlobe to the tip of the nose. The specimens were collected in normal saline and stored at -80 °C. Frozen nasopharyngeal swab samples were taken and naturally thawed at room temperature. Two hundred microliters of the sample was extracted, and ADV-DNA replication was detected by using a RT‒PCR kit (Qiagen, USA). According to the kit's instructions, the bronchoalveolar lavage fluid (BALF) sample was used for the DNA extraction. Commercial primers and probes were utilized for PCR amplification. Furthermore, ADV DNA > 1.0 × 10^3^ copies/mL was considered to be a positive result.

### Statistical analysis

SPSS25.0 software was applied for the data analysis. Normality was analyzed via the Shapiro‒Wilk method. Normally, quantitative data are expressed as the mean ± standard deviation (x ± s). The independent sample t test was utilized for comparisons between the two groups [12]. Non-normally distributed quantitative data are shown as the median (IQR, interquartile range). The rank-sum test was used for the comparisons between the groups. In addition, a percentage (%) was utilized to analyze enumeration data, and the χ2 test was performed. Logistic regression was also performed [13]. *P* < 0.05 and *P* > 0.1 were used as independent variable inclusion and exclusion criteria, respectively, and the test level was set at 0.05 (both sides). R3.6.3 was utilized to build a nomogram risk prediction model. Moreover, diagnostic performance was evaluated by using receiver operating characteristic (ROC) curves. For the calibration, a curve was drawn. To assess the model's fit, we used the Hosmer–Lemeshow (H–L) goodness of fit test. Furthermore, a *P* value < 0.05 was considered to be statistically significant.

## Results

### Comparisons of clinical characteristics of pediatric patients with ADVP in the PIBO and non-PIBO groups

A total of 863 cases with ADVP were enrolled in the study, including 46 cases (5.3%) and 817 cases (94.7%) in the PIBO and non-PIBO groups, respectively. A statistically significant difference was found between the PIBO and non-PIBO groups, with 35 men (76.1%) and 525 men (64.3%) in each group, respectively (*P* = 0.1, Table 1). The median age at the time of diagnosis of PIBO was younger, but there was no significant (*P* = 0.55, Table 1). Moreover, the age of onset in the two groups was mainly concentrated between 6–24 months. There was a significantly longer hospital stay (*P* = 0.02) for patients with PIBO, as well as a higher duration of fever (*P* = 0.00, Table 1). Additionally, wheezing, lung consolidation, and complications were also more frequent among patients in the PIBO group (*P* < 0.05, Table 1). The percentage of neutrophils (N%, *P* = 0.000), lactate dehydrogenase (LDH, *P* = 0.000), D-dimer (DD, *P* = 0.000), ADV load (*P* = 0.026), alanine transaminase (ALT, *P* = 0.000) and aspartate aminotransferase (AST, *P* = 0.000) were significantly higher in the PIBO group compared to the non-PIBO group. However, platelet (PLT, *P* = 0.02) was the opposite of that. Except for these parameters, there were no significant differences in allergic constitution, mixed infection, leukocyte (WBC), hemoglobin (Hb), C-reactive protein (CRP), procalcitonin (PCT), fibrinogen (Fig), creatine kinase isoenzyme (CK-MB), total immunoglobulin (Ig) A, IgG, IgM, CD3 + , CD3 + CD4 + , and CD3 + CD8 + . In addition, the PIBO group was more likely to receive interventions such as IMV, corticosteroids, IV immunoglobulin, and tracheoscopy (*P* = 0.000, Table 1).

**Table 1:** Comparisons of clinical characteristics of pediatric patients with ADVP in the PIBO and non-PIBO groups

Variables	Non-PIBO group (*n* = 817)	PIBO group (*n* = 46)	*P* Value
**Characteristic**			
Male, *n* (%)	525 (64.3)	35 (76.1)	0.1
Age [M (P25-P75)]/(months)	13 (11–24)	12 (11–24)	0.55
**Age group (months),** ***n*** **(%)**			
< 6	55 (6.7)	3 (6.5)	0.96
6–24	560 (68.5)	34 (73.9)	0.44
25–36	202 (24.7)	9(19.6)	0.43
Allergic constitution [*n* (%)]	162 (19.8)	13 (28.3)	0.17
**Sigs and symptoms**			
Median duration of hospitalization [M (P25-P75)]/d	7 (5–10)	9(6–13)	0.02
Duration of fever [M (P25-P75)]/d	7 (5–11)	18 (11–24)	0.000
Wheezing [*n* (%)]	163(20.0)	15(32.6)	0.04
**Laboratory characteristic**			
WBC [M (P25-P75)]/ × 10^9^ L^−1^	7.3 (5.4–10.3)	7.2 (5.5–9.7)	0.1
N% [M (P25-P75)]/%	42.1 (26.8–55.9)	58.6(48.7–69.9)	0.000
Hb [M (P25-P75)]/g·L^−1^	111(104–118)	109 (97–117)	0.36
PLT [M (P25-P75)]/ × 10^9^ L^−1^	314 (228–417)	250 (161–313)	0.02
CRP [M (P25-P75)]/mg·L^−1^	6.5 (3.1–17.9)	9.8 (3.1–34.5)	0.10
PCT [M (P25-P75)]/ng·mL^−1^	0.2 (0.07–0.9)	0.5 (0.2–1.3)	0.95
LDH [M (P25-P75)]/U·L^−1^	382 (307–517)	587 (460–768)	0.00
Fig [M (P25-P75)]/mg·dL^−1^	3.1 (2.4–4.0)	2.5 (2.0–3.1)	0.98
DD [M (P25-P75)]/mg·L^−1^	0.7 (0.4–1.6)	2.0 (0.8–3.3)	0.026
CK-MB [M (P25-P75)]/ng·mL^−1^	29 (22–42)	34 (26–45)	0.81
ALT [M (P25-P75)]/U·L^−1^	19 (14–30)	29 (20–62)	0.00
AST [M (P25-P75)]/U·L^−1^	45(36–59)	90 (61–111)	0.00
Mixed infection [*n* (%)]	564 (69.0)	34 (73.9)	0.49
ADV load from BALF, log_10_ copies/mL	6.0 (4.3–7.3)	6.4 (4.9–7.4)	0.026
**Humoral immunity [M (P25-P75), %]**			
IgG [M (P25-P75)]/g·L^−1^	7.8 (6.4–9.6)	8.1 (5.9–11.5)	0.72
IgA [M (P25-P75)]/g·L^−1^	0.7 (0.5–1.2)	0.9 (0.5–1.3)	0.99
IgM [M (P25-P75)]/g·L^−1^	1.0 (0.7–1.3)	0.9 (0.6–1.3)	0.6
**Cellular immunity [M (P25-P75), %]**			
CD3^+^	52.2 (43.4–60.1)	57.1 (45.9–63.2)	0.17
CD3^+^ CD4^+^	26.4 (19.9–34.4)	26.3 (19.6–36.5)	0.58
CD3^+^ CD8^+^	20.9(17.2–26.4)	22.7 (17.5–31)	0.23
Lung consolidation [*n* (%)]	302 (37.0)	27(58.7)	0.003
Complication [*n* (%)]	192 (23.5)	39 (84.8)	0.000
**Treatment,** ***n*** **(%)**			
IMV	38 (4.7)	30 (65.2)	0.000
Corticosteroids	230 (28.2)	32 (69.6)	0.000
IV immunoglobulin	326 (39.9)	36 (78.3)	0.000
Tracheoscopy intervention therapy	245 (30.0)	29 (63.0)	0.000

*ADV* Adenovirus, *ADVP* Adenovirus pneumonia, *PIBO* Post-infectious bronchiolitis obliterans, *WBC* Leukocyte, *N%* Percentage of neutrophils, *Hb* Hemoglobin, *PLT* Platelet, *CRP* C-reactive protein, *PCT* Procalcitonin, *LDH* Lactate dehydrogenase, *Fig* Fibrinogen, *DD* D-dimer, *CK-MB* Creatine Kinase Isoenzyme, *ALT* Gluten Laboratory data of Alanine transaminase, *AST* Aspartate aminotransferase, *IgG* Immunoglobulin G, *IgM* Immunoglobulin M, *IgA* Immunoglobulin A, *IMV* Invasive mechanical ventilation

### Comparisons of clinical characteristics of pediatric patients with severe ADVP after IMV in the PIBO and non-PIBO groups

**Fig. 1:**
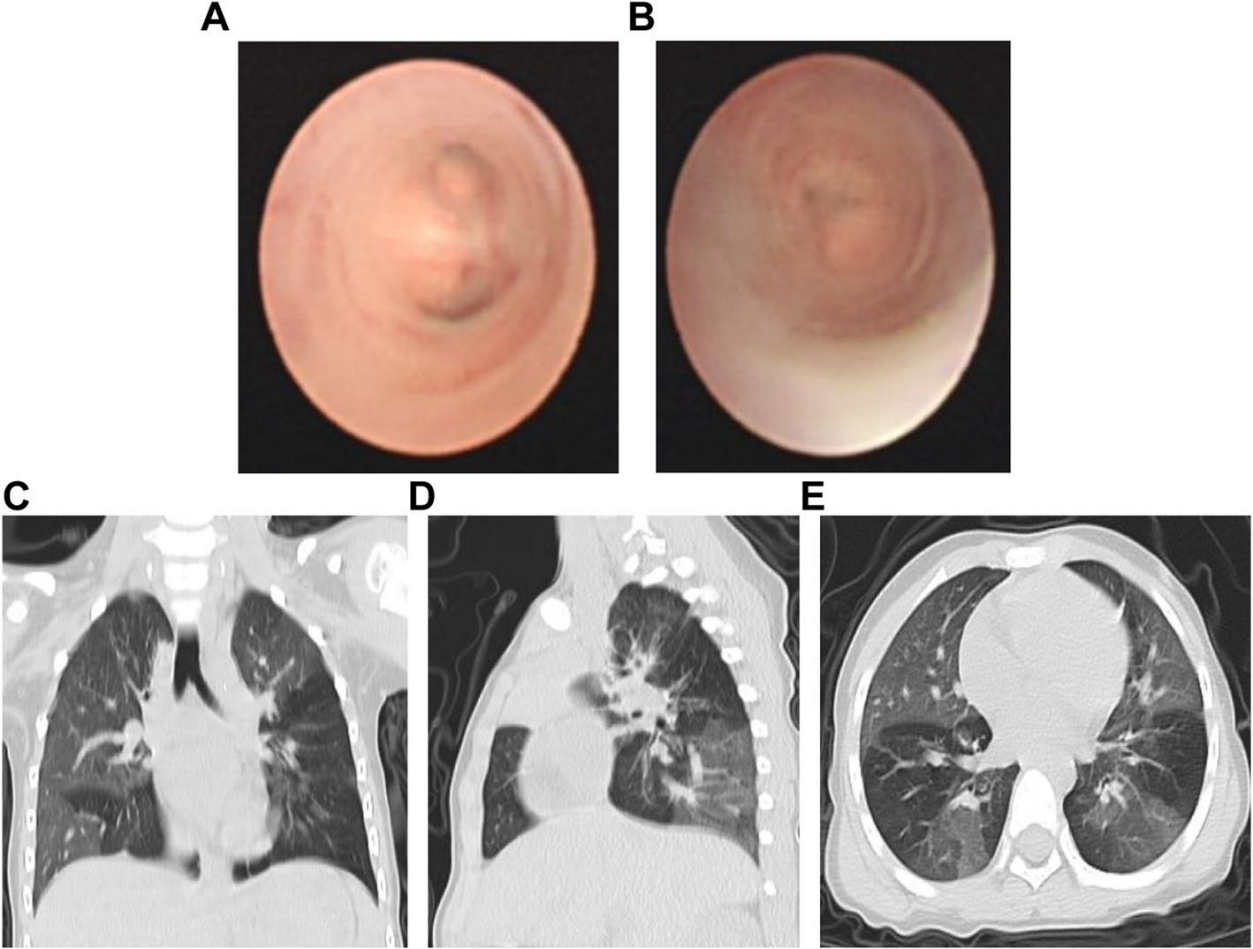
The bronchoscopic and imaging features of a child with ADVP after IMV who developed PIBO 2 years later. **A**-**B** Different degrees of middle and small bronchial lumen occlusion and fibrous plate hyperplasia were observed by using fiberoptic bronchoscopy; **C**-**D** chest radiographs of the patient from the posterior and lateral views showed that permeability of both lungs was enhanced; **E** mosaic perfusion sign was observed by using a CT slice

Due to the high proportion of PIBO in children with ADVP after IMV, we examined the causes of PIBO in cases of ADVP after IMV. There were 66 cases of ADVP after IMV, including PIBO (33 cases, 50.0%) and non-PIBO (33 cases, 50.0%) groups. There were 29 men (87.9%) and 21 men (63.6%) in the PIBO and non-PIBO groups, respectively. Moreover, the difference was statistically significant (*P* = 0.02, Table 2). There was no significant difference in age between the PIBO group and the non-PIBO group (*P* = 0.88, Table 2). Additionally, patients in the PIBO group had a longer duration of IMV treatment (*P* = 0.03) and duration of fever (*P* = 0.00) throughout the disease (Table 2). Significant increases in mixed fungi coinfection (*P* = 0.006) were observed in the PIBO group (Table 2). The median ADV load of the PIBO group was higher than that of the non-PIBO group (7.1 vs. 6.0, respectively, *P* = 0.002, Table 2). Furthermore, only one child with PIBO (8 months) died due to recurrent infection, while no child in the non-PIBO group died. Figure 1 shows the bronchoscopic and imaging features of a child with ADVP after IMV who developed PIBO 2 years later.

**Table 2:** Comparisons of clinical characteristics of pediatric patients with severe ADVP after IMV in the PIBO and non-PIBO groups

Variables	Non-PIBO group (*n* = 33)	PIBO group (*n* = 33)	*P* Value
**Characteristic**			
Male, *n* (%)	21 (63.6)	29 (87.9)	0.02
Age [M (P25-P75)]/(months)	12 (8–24)	12 (10–22)	0.88
**Age group (months),** ***n*** **(%)**			
< 6	3 (9.1)	2 (6.1)	0.64
6–24	24 (72.7)	28 (84.8)	0.23
25–36	6 (18.2)	3 (9.1)	0.28
**Significant comorbidities,** ***n*** **(%)**			
Premature birth	5 (15.2)	3 (9.1)	0.45
A history of wheezing	15 (45.5)	14 (42.4)	0.80
Congenital heart disease	5 (15.2)	3 (9.1)	0.45
Allergic constitution [*n* (%)]	10 (30.3)	12 (36.4)	0.1
**Sigs and symptoms**			
Median duration of hospitalization [M (P25-P75)]/d	24 (17–27)	27 (23–32)	0.01
Duration of fever [M (P25-P75)]/d	20 (15–22)	23 (20–31)	0.00
Wheezing [*n* (%)]	19 (57.6)	10 (30.3)	0.03
**Laboratory characteristic**			
WBC [M (P25-P75)]// × 10^9^ L^−1^	6.5 (4.2–9.9)	6.4 (4.4–10.6)	0.57
N% [M (P25-P75)]/%	62.5 (51.1–70.4)	64.3 (47.1–71.1)	0.33
Hypohemia, *n* (%)	24 (72.7)	28 (84.8)	0.23
PLT [M (P25-P75)]/ × 10^9^ L^−1^	285 (197–445)	311 (189–428)	0.84
CRP [M (P25-P75)]/mg·L^−1^	8.5 (3.1–23.3)	9 (3.1–18.1)	0.36
PCT [M (P25-P75)]/ng·mL^−1^	0.9 (0.06–1.9)	1.0 (0.42–1.91)	0.28
LDH [M (P25-P75)]/U·L^−1^	544 (351–890)	687 (464–968)	0.29
Fig [M (P25-P75)]/mg·dL^−1^	2.1 (1.6–2.9)	2.3 (1.6–3.0)	0.29
DD [M (P25-P75)]/mg·L^−1^	2.2(1.6–3.1)	2.3 (1.7–2.9)	0.14
CK-MB [M (P25-P75)]/ng·mL^−1^	42 (27–56)	36 (28–45)	0.48
ALT [M (P25-P75)]/U·L^−1^	40.8 (18.4–75.4)	32.4 (20.2–70.8)	0.33
AST [M (P25-P75)]/U·L^−1^	84 (60–168)	98 (70.3–142)	0.30
Mixed infection [*n* (%)]	29(87.9)	33 (100)	0.04
Bacterial coinfection [*n* (%)]	18 (54.5)	22 (66.7)	0.31
MP-coinfection [*n* (%)]	10 (30.3)	13 (41.2)	0.44
Virus-coinfection [*n* (%)]	12 (36.4)	24 (72.7)	0.11
Fungi coinfection [*n* (%)]	14 (42.4)	25 (75.8)	0.006
ADV load from BALF, log_10_ copies/mL	6.0 (5–6.9)	7.1 (6.4–8.1)	0.002
**Humoral immunity [M (P25-P75), %]**			
IgG [M (P25-P75)]/g·L^−1^	8.4 (6.9–13.4)	8.0 (6.7–10.1)	0.24
IgA [M (P25-P75)]/g·L^−1^	0.8 (0.6–1.1)	0.8 (0.6–1.2)	0.78
IgM [M (P25-P75)]/g·L^−1^	0.7 (0.3–1.0)	0.7 (0.4–1.02)	0.54
**Cellular immunity [M (P25-P75), %]**			
CD3^+^	50.4 (43.1–57.7)	50.5 (40.4–59.4)	0.76
CD3^+^ CD4^+^	20.0 (15.7–30.7)	22.4 (18.7–27.8)	0.59
CD3^+^ CD8^+^	21.5 (16.4–24.8)	20.2 (15.6–28.7)	0.65
**Radiological characteristics [*n*** **(%)]**			
Lung consolidation	19 (57.6)	25 (75.8)	0.12
Pleural effusion	5 (15.2)	7 (21.2)	0.52
Associated multy organ dysfunction, *n* (%)	30 (90.9)	33 (100)	0.08
**Treatment,** ***n*** **(%)**			
Length of IMV[M(P25-P75)]/d	8 (7–12)	11 (9–14)	0.03
CRRT, *n* (%)	4 (12.1)	4 (12.1)	1.00
ECMO, *n* (%)	0 (0)	1 (3.0)	0.31
Corticosteroids	23 (69.7)	28 (84.8)	0.14
IV immunoglobulin	29 (87.9)	31 (93.9)	0.39
Tracheoscopy intervention therapy	14 (42.4)	21 (63.6)	0.08
Death	0	1	0.31

*ADV* Adenovirus, *ADVP* Adenovirus pneumonia, *IMV* Invasive mechanical ventilation, *PIBO* Post-infectious bronchiolitis obliterans, *WBC* Leukocyte, *N%* Percentage of neutrophils, *Hb* Hemoglobin, *PLT* Platelet, *CRP* C-reactive protein, *PCT* Procalcitonin, *LDH* Lactate dehydrogenase, *Fig* Fibrinogen, *DD* D-dimer, *CK-MB* Creatine Kinase Isoenzyme, *ALT* Gluten Laboratory data of Alanine transaminase, *AST* Aspartate aminotransferase, *MP* Mycoplasma, *IgG* Immunoglobulin G, *IgM* Immunoglobulin M, *IgA* Immunoglobulin A, *CRRT* Continuous renal replacement therapy support, *ECMO* Extracorporeal membrane oxygenation support

### Predictive impact indicators for PIBO

Univariate logistic regression revealed that duration of fever (odds ratio [OR]: 1.088; 95% CI: 1.031–1.148), IMV (OR: 7.597; 95% CI: 2.900–19.901), complications (OR: 3.921; 95% CI: 1.539–9.989), and N% (OR: 1.045; 95% CI: 1.016–1.074) were independent impact indicators for PIBO in pediatric patients with ADVP (*P* < 0.05, Table 3).

**Table 3:** Logistic regression analysis of objective indicators of PIBO formation

Variable	Partial regression coefficient (*β*)	*SE*	Wald *χ*2	*P*	OR	95% CI
Duration of fever	0.085	0.028	9.444	0.002	1.088	1.031–1.148
IMV	2.028	0.491	17.032	0.00	7.597	2.900–19.901
Complication [*n*(%)]	1.366	0.477	8.203	0.004	3.921	1.539–9.989
N%	0.044	0.014	9.450	0.002	1.045	1.016–1.074
Constant	-5.153	1.016	25.706	0	0.006	

*PIBO* Post-infectious bronchiolitis obliterans, *SE* Standard error, *OR* Odds ratio, *CI* Confidence interval, *IMV* Invasive mechanical ventilation, *N%* Percentage of neutrophils

### Predictive impact indicators for PIBO (IMV treatment)

To examine the formation of PIBO in ADVP cases with IMV, we performed an impact factor analysis for PIBO. Male sex (OR: 0.186, 95% CI: 0.036–0.973), duration of fever (OR: 1.151; 95% CI: 1.017–1.302), ADV load (OR: 1.842; 95% CI: 1.129–3.006), and fungi coinfection (OR: 5.616; 95% CI: 1.369–23.041) were independent impact factors for the formation of PIBO (*P* < 0.05, Table 4).

**Table 4:** Logistic regression analysis of objective indicators about PIBO formation after IMV

Variable	Partial regression coefficient (*β*)	*SE*	Wald *χ*2	*P*	OR	95% CI
Male	-1.680	0.843	3.968	0.046	0.186	0.036–0.973
Duration of fever	0.140	0.063	4.962	0.026	1.151	1.017–1.302
ADV load from BALF	0.611	0.250	5.980	0.014	1.842	1.129–3.006
Fungi coinfection	1.726	0.720	5.741	0.017	5.616	1.369–23.041
Constant	-5.943	2.302	6.666	0.010	0.003	

*PIBO* Post-infectious bronchiolitis obliterans, *IMV* Invasive mechanical ventilation, *SE* Standard error, *OR* Odds ratio, *CI* Confidence interval, *ADV* adenovirus

### A nomogram prediction model of PIBO occurrence was constructed in pediatric patients with ADVP after IMV

**Fig. 2:**
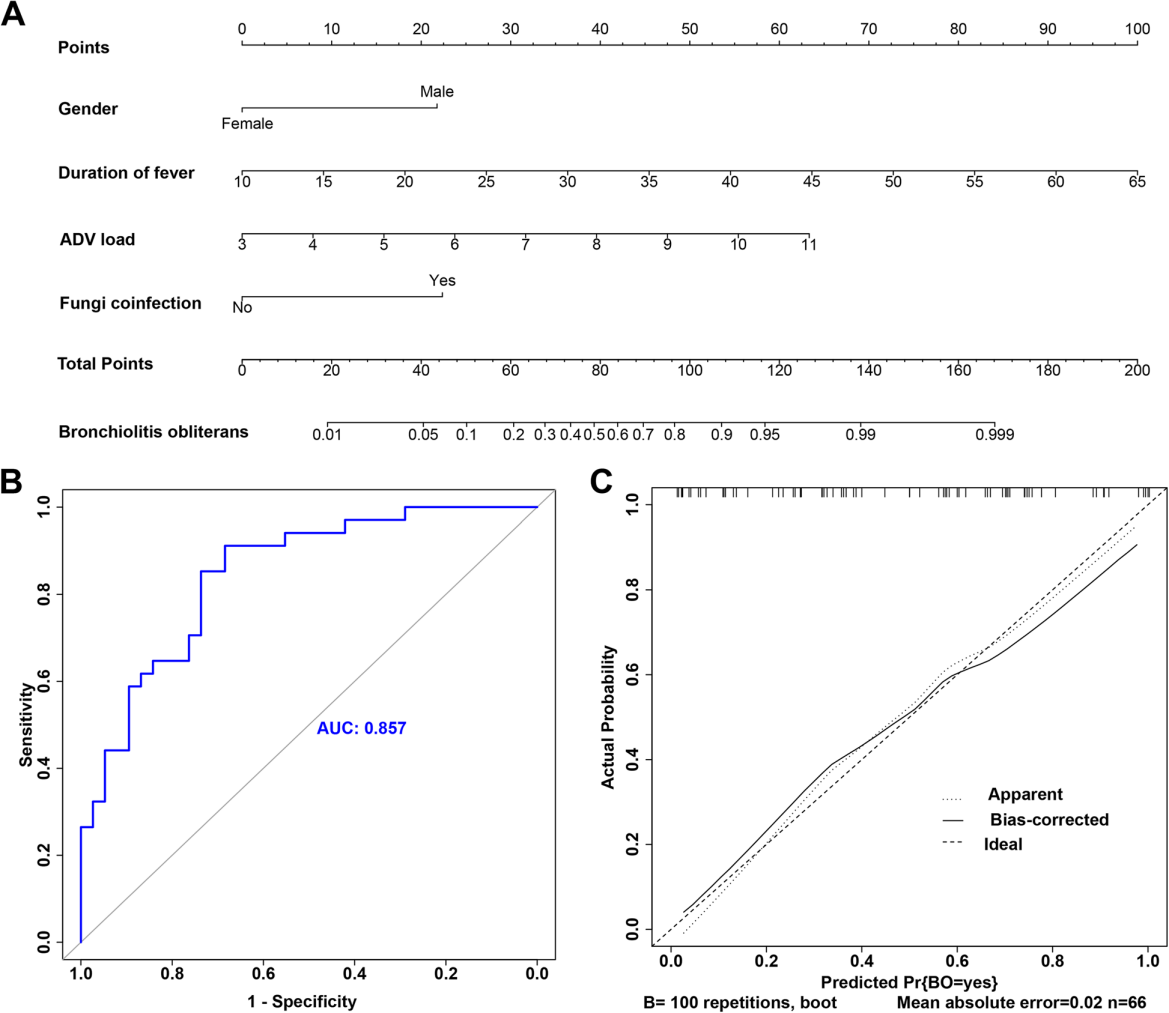
Construction and analysis of the nomogram prediction model for PIBO. **A** The nomogram; In this model, the variables of gender, duration of fever, ADV load, and fungi coinfection had corresponding points on the corresponding axes in the column line graph. The value corresponding to the vertical line of the point (upward) on the scoring scale was the score of that variable, and the total score was obtained by adding the scores corresponding to the above four variables. A vertical line was drawn down from the "total score" line to calculate the probability of PIBO in patients with ADVP. B ROC curve analysis; C calibration curve analysis

As shown in Fig. 2A, based on the abovementioned four impact factors (gender, duration of fever, ADV load, and fungi coinfection), the PIBO formation nomogram prediction model of children with ADVP under IMV was established. The ROC curve for the risk of PIBO occurrence was used to assess the discrimination of the nomogram model. The calculated AUC by using the ROC model to predict impact factors for PIBO in children with ADVP under IMV was 0.857 (95% CI: 0.744–0.928), thus suggesting good discrimination from the nomogram model (Fig. 2B). Subsequently, a calibration curve was drawn, and the H–L goodness-of-fit test was performed to assess the accuracy of the nomogram model. The calibration curve was a straight line with a slope close to 1 (Fig. 2C), thus indicating that the model predicted that the risk of PIBO was in good agreement with the actual risk. The H–L goodness-of-fit was 68.75 (*P* < 0.01), thus indicating good calibration for the model.

## Discussion

Through a retrospective study of 863 children under 3 years of age with ADVP at our Children's Medical Center in 2019, we found that IMV, duration of fever, complications, and N% were the risk indicators for PIBO in children with ADVP. More importantly, we further analyzed the risk indicators for PIBO in children with severe ADVP after IMV and successfully constructed a nomogram that can screen which children are at high risk of developing PIBO. The nomogram could be used as a clinical reference to predict the risk rate of developing PIBO in children with severe ADVP after IMV and provide a basis for further prevention and treatment of PIBO.

Nomogram is an individualized, visualized statistical prediction model based on cox or logistic regression models [14]. The model has been widely used to assess disease risk and prognosis [15]. Wang et al. constructed a nomogram to predict the probability of psychosocial and behavioral problems in children and adolescents from an online questionnaire involving 12,186 children (6–11 years) and adolescents (12–16 years) [16]. Zhang et al. retrospectively analyzed the clinical baseline characteristics of 64 children in China and further created a recurrence risk model of children Neurophyllitis optica spectrum disorders using a nomogram [17]. Cheng et al. analyzed the clinical characteristics and risk factors for the development of PIBO in children (0–14 years old) with refractory Mycoplasma pneumoniae pneumonia (MPP) and constructed a nomogram to predict the risk ratio for the development of PIBO [18]. Similarly, the current study analyzed the risk factors for the development of PIBO in 66 children (0–36 months) with ADVP after IMV retrospectively and constructed a nomogram. The AUC of the model for predicting the risk of PIBO in children with ADVP after IMV was 0.857, thus indicating that the nomogram model had good discrimination. The calibration curve was a straight line with a slope close to 1. Furthermore, the H–L goodness-of-fit test showed that the nomogram model had a good degree of calibration.

Zhong et al. revealed that IMV affects PIBO in ADVP [9]. Xie et al. also found that duration of fever was an influencing indicator for PIBO in children with ADVP [19]. Complications suggest that ADVP is more likely to progress to severe pneumonia, the course of the disease is prolonged [20, 21], and the possibility of PIBO is increased. N% may be associated with acute inflammation [22, 23]. In a study on risk factors of PIBO in children with ADVP in Jilin city, China, N% in the PIBO group was higher than that in the non-PIBO group [2]. Similar to the above studies, the present study found IMV, duration of fever, and complications were risk indicators for PIBO in children with ADVP. In addition, our study found that N% was also the risk factor for the development of PIBO in children with ADVP, which is one of the innovative points of our study.

This study found that most IMV children with ADVP were male (87.9%), which was consistent with data from previous studies [24]. Furthermore, ADV load may be associated with the severity of pneumonia and the risk of developing PIBO [25]. Children with severe ADVP who had mixed infections accounted for 62 cases (93.9%) in this study. We speculated that children with severe ADVP might be prone to fungal coinfections after long-term application of IMV and antibiotics. Fungal coinfections can further aggravate the lung injury caused by ADVP.

Cytokine profiles can help advance understanding of how/what/why the harmful response that damages the respiratory tissues in such an intense manner [1, 26, 27]. For instance, Hou et al. showed that cytokines, such as IL-2, IL-6, IL-10, TNF-α, and IFN-γ, could help determine the severity of ADVP and could be used as an objective indicator to evaluate the prognosis of severe ADVP in pediatric patients [27]. Zheng et al. clarified that IL-6, IL-10, and IFN-γ levels were significantly higher with severity in patients with ADVP [26]. The current study found that neutrophil percentage (N%, *P* = 0.000) and lactate dehydrogenase (LDH, *P* = 0.000) were significantly higher in the PIBO group compared to the non-PIBO group, suggesting that N% and LDH indicators might be closely related to PIBO formation in children with ADVP. However, our manuscript was a retrospective study of 863 cases of ADVP. The sample size was large. Also, cytokine profiles testing is not a routine test like blood tests. Therefore, the current study does not include information on "cytokines profiles," which was a limitation of our study. In future studies, we will examine some cytokines to help elucidate their role in the pathogenesis of ADVP.

Indeed, the majority of children who develop PIBO are under 2 years of age, which in part, due to the immature small airways children younger than two years of age have [10]. Similar to previous studies [10], in the current study, the proportion of children under 2 years of age was as high as 73.9% of 863 children with ADVP. In a study of 187 children with ADVP (10 months to 12 years), Yu et al. found that half of the patients with severe ADVP were < 2 years old, and the median age of children who developed PIBO was 16.5 (IQR: 11–25.25 months) months [2]. The study by Colom et al. included 109 cases and 99 controls in children under 3 years of age. Among them, the median age of children who developed PIBO was 6 (IQR: 1–26 months) months [28]. Similar to the above study, we selected children with ADVP aged 0–36 months as our study population.

Colom et al. found that both ADV infection and IMV were strongly associated with the development of PIBO in Argentine children with ADVP under the age of 3 years [28]. In contrast to the study by Colom et al. [28], our study found that IMV, duration of fever, complications, and N% were the main risk factors for the development of PIBO in children with ADVP through the observation and analysis of clinical baseline information in 863 children with ADVP under the age of 3 years. More importantly, our study further analyzed the occurrence of PIBO in children with ADVP after IMV and constructed a nomogram to predict the risk ratio for PIBO. Cheng et al. constructed a nomogram model for the occurrence of PIBO by analyzing the data of 141 children with MPP (aged 1–14 years) [18]. Unlike the study by Cheng et al. [18], our study was conducted on children with ADVP aged 0–3 years and not on children with MPP aged 1–14 years. In addition, to our knowledge, our study was the first to construct a nomogram for predicting the risk of developing PIBO in children with ADVP who have experienced IMV. However, there are some limitations in our study. This study was a single-center study and indicates that the Hunan region does not represent other regions of China. Therefore, we hope that multicenter studies will be performed in the future. In addition, this study included mixed infections, which may have affected the prognosis of PIBO.

In conclusion, we have developed a nomogram that could be applied to predict the risk ratio of developing PIBO in children with ADVP after IMV. Our study might provide a basis for further advancement in the prevention and treatment of PIBO.

## Data Availability

All data generated or analyzed during this study are included in this article. Further enquiries can be directed to the corresponding author.
